# Medical Home Implementation and Follow-Up of Cancer-Related Abnormal Test Results in the Veterans Health Administration

**DOI:** 10.1001/jamanetworkopen.2024.0087

**Published:** 2024-03-14

**Authors:** Suja S. Rajan, Shashank Sarvepalli, Li Wei, Ashley N. D. Meyer, Daniel R. Murphy, Debra T. Choi, Hardeep Singh

**Affiliations:** 1 Department of Management, Policy & Community Health, School of Public Health, The University of Texas Health Science Center at Houston; 2 Department of Medicine, Baylor College of Medicine, Houston, Texas; 3 Center for Innovations in Quality, Effectiveness and Safety (IQuESt), Michael E. DeBakey Veterans Affairs Medical Center, Houston, Texas

## Abstract

**Importance:**

Lack of timely follow-up of cancer-related abnormal test results can lead to delayed or missed diagnoses, adverse cancer outcomes, and substantial cost burden for patients. Care delivery models, such as the Veterans Affairs’ (VA) Patient-Aligned Care Team (PACT), which aim to improve patient-centered care coordination, could potentially also improve timely follow-up of abnormal test results. PACT was implemented nationally in the VA between 2010 and 2012.

**Objective:**

To evaluate the long-term association between PACT implementation and timely follow-up of abnormal test results related to the diagnosis of 5 different cancers.

**Design, Setting, and Participants:**

This multiyear retrospective cohort study used 14 years of VA data (2006-2019), which were analyzed using panel data-based random-effects linear regressions. The setting included all VA clinics and facilities. The participants were adult patients who underwent diagnostic testing related to 5 different cancers and had abnormal test results. Data extraction and statistical analyses were performed from September 2021 to December 2023.

**Exposure:**

Calendar years denoting preperiods and postperiods of PACT implementation, and the PACT Implementation Progress Index Score denoting the extent of implementation in each VA clinic and facility.

**Main Outcome and Measure:**

Percentage of potentially missed timely follow-ups of abnormal test results.

**Results:**

This study analyzed 6 data sets representing 5 different types of cancers. During the initial years of PACT implementation (2010 to 2013), percentage of potentially missed timely follow-ups decreased between 3 to 7 percentage points for urinalysis suggestive of bladder cancer, 12 to 14 percentage points for mammograms suggestive of breast cancer, 19 to 22 percentage points for fecal tests suggestive of colorectal cancer, and 6 to 13 percentage points for iron deficiency anemia laboratory tests suggestive of colorectal cancer, with no statistically significant changes for α-fetoprotien tests and lung cancer imaging. However, these beneficial reductions were not sustained over time. Better PACT implementation scores were associated with a decrease in potentially missed timely follow-up percentages for urinalysis (0.3–percentage point reduction [95% CI, −0.6 to −0.1] with 1-point increase in the score), and laboratory tests suggestive of iron deficiency anemia (0.5–percentage point reduction [95% CI,−0.8 to −0.2] with 1-point increase in the score).

**Conclusions and Relevance:**

This cohort study found that implementation of PACT in the VA was associated with a potential short-term improvement in the quality of follow-up for certain test results. Additional multifaceted sustained interventions to reduce missed test results are required to prevent care delays.

## Key Points

**Question:**

Is the implementation of the Patient-Aligned Care Team (PACT) initiative associated with timely follow-up of abnormal test results related to the diagnosis of 5 different cancers?

**Findings:**

This cohort study analyzing 6 data sets representing 5 different types of cancers found that implementation of PACT in the Veterans Health Administration only had a short-term association with reduction in potentially missed timely follow-ups of abnormal test results for most cancer-related diagnostic tests.

**Meaning:**

These findings suggest that additional multicomponent and sustained interventions are needed to address problems related to missed follow-up of abnormal test results, which is an intractable problem to solve in most health systems.

## Teaser

This cohort study investigates whether there is a long-term association between the Veterans Affairs (VA) Patient-Aligned Cancer Team implementation and timely follow-up of abnormal diagnostic test results for 6 different cancers.

## Introduction

Lack of timely follow-up of cancer-related abnormal test results can lead to delayed or missed diagnoses, increased risk of disease progression, and consequently advanced stage at diagnosis.^1,2,3,4,5,6^ Advanced cancer stage at diagnosis is often associated with poor prognosis and higher costs.^5,6,7,8,9^ Missed or delayed opportunities to act on cancer-related abnormal test results remain common.^10,11,12,13,14,15,16,17,18,19^ Up to 38% of patients with a lung cancer diagnosis had missed or delayed follow-up opportunities,^12^ and a similar percentage of missed or delayed follow-up opportunities occurred among patients with breast, colorectal, hepatocellular, or bladder cancer.^11,16,18,19^ These studies highlight the need to streamline outpatient care processes to ensure timely follow-up of test results.

Reliable follow-up of abnormal test results is often dependent on coordination between care teams and other workflow factors.^20^ Implementation of care delivery models such as the patient-centered medical home (PCMH), which emphasize care coordination, might improve follow-up of abnormal test results.^21,22,23^ Implementation of PCMH models has shown mixed results related to costs, but it has generally been associated with better quality of care, and patient and caregiver experience.^21,22,23,24,25,26,27^

The Department of Veterans Affairs’ (VA) PCMH initiative, called the Patient-Aligned Care Team (PACT), was implemented nationally in the VA facilities and clinics between 2010 and 2012.^25^ This initiative cost the VA approximately $2 billion and required intensive national, regional, and local participation to support this effort. Performance-related metrics were also enforced to assess PACT’s implementation.^28^ Because VA facilities varied in the extent to which various PACT components were implemented, the extent of PACT implementation was measured using the PACT Implementation Progress Index (Pi^2^) Score*.* Past studies have reported that the PACT implementation was associated with improvements in clinical processes, quality of care, and patient outcomes. However, evaluations of long-term associations between PACT and timely follow-up of abnormal test results are still needed.

We evaluated the long-term association between PACT implementation and timely follow-up of abnormal test results related to the diagnosis of 5 different cancers. We also examined the association between the extent of PACT implementation at each facility and timely follow-up of abnormal test results. We hypothesized that PACT implementation and the extent of PACT implementation would be associated with reduced percentage of potentially missed timely follow-up of abnormal test results.

## Methods

### Study Design and Data Source

We conducted a retrospective observational cohort study using data from the VA’s national Corporate Data Warehouse (CDW). The CDW contains clinical data for more than 9 million veterans seen in the inpatient and outpatient settings in more than 1000 VA facilities nationwide. A facility in this study was defined as a VA inpatient facility, such as a hospital, or outpatient facility, such as a community-based outpatient clinic (CBOC), where a patient health care encounter occurs.

We evaluated variations in percentage of potentially missed timely follow-up of 6 different cancer-related abnormal diagnostic test results over a 14-year period (2006-2019), thereby examining the differences in these percentages before and after the PACT implementation from 2010 to 2012. The 6 diagnostic tests studied were: (1) urinalysis for evaluating bladder cancer; (2) mammograms for evaluating breast cancer; (3) fecal occult blood test (FOBT) or fecal immunohistochemical test (FIT) for evaluating colorectal cancer; (4) laboratory tests indicating iron deficiency anemia (IDA) for evaluating colorectal cancer; (5) α-fetoprotein testing for evaluating hepatocellular carcinoma (HCC); and (6) lung imaging for evaluating lung cancer.

We used previously developed electronic triggers (e-triggers) to mine vast amounts of CDW data to identify follow-up delays and missed opportunities after an abnormal test result.^10,16,17,18,19,29,30,31,32,33,34^ Details about the types of cancer diagnostic tests considered, the criteria for identifying abnormal test results for each of these diagnostic tests, the criteria for exclusion, and the criteria for timely follow-up are described in detail in Table 1 and in previously published studies.^10,16,17,18,19,30,33,34^ Patient records flagged by the e-trigger are highly likely to have a missed opportunity for follow-up after an abnormal test (positive predictive values for missed follow-up ranged from 56% to 82%). However, because we did not conduct medical record audits to confirm presence or absence of follow-up in the current study, we used the term “potentially missed” in this study. We first applied e-trigger algorithms to the CDW to identify abnormal results for the 6 types of tests during the 14-year study period. The algorithm query then programmatically scanned a previously validated follow-up period ranging from 30 days to 7 months after the abnormal test result, depending on the test, to identify records with absence of appropriate and timely follow-up. Patients were excluded if they died or did not need follow-up evaluation due to terminal illnesses (based on *International Classification of Diseases, Ninth Revision [ICD-9]* and *ICD-10 *codes) within the predetermined follow-up period. Based on the algorithm’s query application in the CDW, if appropriate follow-up was missing, tests with abnormal results were flagged as “e-trigger positive.”

**Table 1.** Criteria for Abnormal Test Results Flagged as e-Trigger Positive Due to Potential Lack of Timely Follow-Up

Cancer diagnostic test under consideration (e-trigger criteria)	Criteria to identify abnormal test results (red flag criteria)	Types of tests and patients excluded after red flag criteria because follow-up is not required for these abnormal test results (clinical exclusion criteria)	Criteria for timely follow-up, which if absent would flag the test as “e-trigger positive” (expected follow-up criteria)
Urinalysis for evaluating bladder cancer	Urinalysis with >50 red blood cells per high-powered field	Any of the following within 1 y prior to the red flag date: bladder cancer diagnosis, terminal illness diagnosis, hospice or palliative care enrollment, cystoscopy performed; any of the following within 3 mo prior to the red flag date: diagnosis of kidney or ureteral stones, potentially hematuria-causing procedure (bladder or prostate biopsy, renal stone surgery, ureteral stent, bladder or kidney surgery); any of the following within 2 d prior to or 7 d after the red flag date: evidence of active UTI (urinalysis or culture consistent with UTI, or antibiotics ordered for UTI); any of the following the red flag date: age <35 y, history of total cystectomy; any of the following within 60 d after the red flag date: deceased, terminal illness diagnosis, hospice or palliative care enrollment	Any of the following within 60 d after the red flag date: urology visit completed, abdominal or pelvic imaging (CT, MRI, ultrasonography), cystoscopy performed, kidney or bladder biopsy, kidney or bladder surgery
Mammograms for evaluating breast cancer	Abnormal mammographic results (BI-RADS 0, 3, 4, or 5)	Any of the following within 1 y prior to the red flag date: breast cancer diagnosis, terminal illness diagnosis, hospice or palliative care, enrollment; any of the following on the red flag date: age <18 y; any of the following within 60 d after the red flag date: deceased, terminal illness diagnosis, hospice or palliative care enrollment	Any of the following within 60 d after (for BIRADS 0, 4, or 5) or within 7 mo after the red flag date (for BIRADS 3), repeat mammography, breast ultrasonography performed, breast MRI performed, breast biopsy performed, breast surgery performed, oncology visit completed
FOBT and FIT for evaluating colorectal cancer	Positive FOBT or FIT result	Any of the following within 3 y prior to the red flag date: colonoscopy performed; any of the following within 1 y prior to the red flag date: colorectal cancer diagnosis, terminal illness diagnosis, hospice/palliative care enrollment, pregnancy (iron-deficiency anemia red flag only); any of the following within 6 mo prior to the red flag date: gastrointestinal bleeding diagnosis (eg, esophageal ulcer), other sources of bleeding (iron-deficiency anemia red flag only); any of the following on the red flag date: age <40 or >75 y, history of total colectomy, history of thalassemia (iron-deficiency anemia red flag only); any of the following within 60 d after the red flag date: deceased, terminal illness diagnosis, hospice/palliative care enrollment, pregnancy (iron-deficiency anemia red flag only)	Any of the following within 60 d after the red flag date: colonoscopy performed, gastroenterology referral performed, multidisciplinary tumor board summary documented
Laboratory tests indicating iron-deficiency anemia for evaluating colorectal cancer	Hemoglobin level ≤11 g/dL, mean corpuscular volume ≤81 fL, and no ferritin ≥100 ng/mL within 12 mo prior to or 60 d after hemoglobin testing (ie, ferritin result <100 or not checked)	Any of the following within 3 y prior to the red flag date: colonoscopy performed; any of the following within 1 y prior to the red flag date: colorectal cancer diagnosis, terminal illness diagnosis, hospice/palliative care enrollment, pregnancy (iron-deficiency anemia red flag only); any of the following within 6 mo prior to the red flag date: gastrointestinal bleeding diagnosis (eg, esophageal ulcer), other sources of bleeding (iron-deficiency anemia red flag only); any of the following on the red flag date: age <40 or >75 y, history of total colectomy, history of thalassemia (iron-deficiency anemia red flag only); any of the following within 60 d after the red flag date: deceased, terminal illness diagnosis, hospice/palliative care enrollment, pregnancy (iron-deficiency anemia red flag only)	Any of the following within 60 d after the red flag date: colonoscopy performed, gastroenterology referral performed, multidisciplinary tumor board summary documented
α-fetoprotein testing for evaluating HCC	α-fetoprotein level >20 ng/mL	Any of the following within 3 y prior to the red flag date: colonoscopy performed; any of the following within 1 y prior to the red flag date: hepatocellular cancer diagnosis gonadal (ovarian or testicular) tumor diagnosis, terminal illness diagnosis, hospice/palliative care enrollment, any of the following within 9 mo prior to the red flag date, pregnancy (diagnosis or positive hCG); any of the following within 6 mo prior to the red flag date: diagnosis of gastrointestinal bleeding (eg, esophageal ulcer), other sources of bleeding (iron-deficiency anemia red flag only); any of the following on the red flag date: age <18 y; any of the following within 60 d after the red flag date: deceased, gonadal (ovarian or testicular) tumor diagnosis, terminal illness diagnosis, hospice/palliative care enrollment, pregnancy (diagnosis or positive hCG)	Any of the following within 60 d after the red flag date: hepatology visit completed, gastroenterology visit completed, surgery visit completed, oncology visit completed, transplant surgery visit completed, multidisciplinary tumor board summary documented, liver imaging (ultrasonography, computed tomography, or magnetic resonance imaging) performed, liver biopsy performed, liver embolization performed, liver surgery performed
Lung imaging for evaluating lung cancer	Chest radiograph or chest CT scan electronically flagged by radiologist as “suspicious for malignancy”	Any of the following on the red flag date: age <18 y; any of the following within 1 y prior to and 30 d after the red flag date: deceased, known lung cancer diagnosis, terminal illness diagnosis, hospice/palliative care enrollment, active TB diagnosis	Any of the following within 30 d after the red flag date: repeat chest radiograph or chest CT scan, PET or PET/CT scan, multidisciplinary tumor board conference, pulmonary visit, cardiothoracic surgery visit; any of the following within 30 d prior to and 30 d after the red flag date: bronchoscopy, lung biopsy, lung surgery

Abbreviations: BI-RADS, Breast Imaging Reporting and Data System; CT, computed tomography; FIT, fecal immunohistochemical test; FOBT, fecal occult blood test; HCC, hepatocellular carcinoma; hCG, human chorionic gonadotropin; MRI, magnetic resonance imaging; PET, positron emission tomography; TB, tuberculosis; UTI, urinary tract infection.

SI conversion factors: To convert hemoglobin to g/L, multiply by 10.0; mean corpuscular volume to μm^3^, multiply by 1.0; ferritin to μg/L, multiply by 1.0; to convert α-fetoprotein to μg/L, multiply by 1.0.

The final analytical data were created at the level of a facility-year by aggregating data at the diagnostic test and patient levels. The dependent and independent variable sections reported here describe in detail the level at which each variable was first extracted and then aggregated to a facility-year level. Six facility-year–level data sets were created, each representing the 6 diagnostic tests previously listed, and were analyzed separately. The facility-year–level data had multiple rows for each facility representing each year under study, and each facility could have up to 14 rows representing a facility-year observation.

This study was approved by Baylor College of Medicine’s institutional review board and the Research and Development Committee at the Michael E. DeBakey VA Medical Center. A waiver of informed consent was granted because it would not be feasible to obtain consent from millions of patients to perform medical record reviews, and the study posed minimal risk to patients. We followed the Strengthening the Reporting of Observational Studies in Epidemiology (STROBE) reporting guideline.

### Dependent Variable

The dependent variable, percentage of e-trigger positive flags, was a continuous variable measuring the percentage of abnormal tests that potentially had a lack of timely follow-up, which were flagged by the e-trigger algorithm per facility per year. The denominator of the *percentage of e-trigger positive flags* variable was the total number of abnormal diagnostic tests in a facility during a year, which should have a follow-up, and the numerator was the number of these abnormal diagnostic tests that potentially lacked timely follow-up. This variable was first created at the diagnostic test–level and then aggregated up to a facility-year level. Reductions in the percentage of e-trigger positive flags imply reductions in missed follow-ups, and if these reductions occur during the years after PACT implementation as compared with the years before PACT implementation, they could potentially indicate improvement in clinical processes associated with PACT.

### Independent Variables

All independent variables were also created separately for each of the 6 diagnostic test types at the facility-year level (Table 2). The variable year of testing was first created at the diagnostic test level, and patient age, gender, race-ethnicity (race and ethnicity analyzed as a single variable), and Nosos score (ie, comorbidity score developed by the VA and used for risk or case-mix adjustment when analyzing VA health care data)^35,36^ were first created at the patient level for each year, and then aggregated up to the facility level for the corresponding year. The remaining variables in Table 2 were extracted at the facility-year or facility level.

**Table 2.** Descriptive Statistics for VA Facilities Identified as Having Diagnostic Tests With Abnormal Results, 2006 to 2019

	No. (%)					
	Bladder cancer urinalysis (facility-year = 12 467; unique facilities = 1306)	Breast cancer mammogram (facility-year = 8000; unique facilities = 1100)	Colorectal cancer FOBT/FIT (facility-year = 13 136; unique facilities = 1307)	Colorectal cancer IDA testing (facility-year = 13 221; unique facilities = 1360)	Hepatocellular cancer α-fetoprotein testing (facility-year = 7421; unique facilities = 1067)	Lung cancer imaging (facility-year = 9108; unique facilities = 1141)
**Dependent variable**						
% Of e-trigger positive flags, mean (SD)	27.8 (26.3)	31.5 (31.0)	55.5 (29.6)	56.0 (28.7)	24.7 (32.8)	45.4 (33.5)
**Independent variables**						
Level of PACT program adoption						
Pi^2^ score, mean (SD)	−0.13 (2.76)^a^	−0.25 (2.81)^a^	−0.15 (2.77)^a^	−0.14 (2.76)^a^	−0.13 (2.67)^a^	−0.15 (2.71)^a^
Sociodemographic characteristics of patients in a facility						
Mean age, mean (SD), y	61.9 (6.4)	54.8 (5.8)	63.6 (4.6)	60.4 (6.7)	61.8 (5.5)	63.6 (5.1)
Gender distribution						
% Of men, mean (SD)	86.9 (18.5)	8.5 (19.1)	95.6 (10.0)	77.3 (24.4)	96.9 (12.4)	95.5 (12.4)
% Of women, mean (SD)	13.1 (18.5)	91.5 (19.1)	4.4 (10.0)	22.7 (24.4)	3.1 (12.4)	4.5 (12.4)
Racial-ethnic distribution						
% Of Hispanic, mean (SD)	4.5 (13.4)	5.1 (15.4)	4.8 (14.4)	5.7 (16.0)	8.4 (21.6)	2.8 (11.5)
% Of non-Hispanic Black, mean (SD)	13.5 (22.2)	20.9 (28.5)	13.2 (21.8)	22.0 (27.9)	26.1 (33.8)	12.3 (22.7)
% Of non-Hispanic White, mean (SD)	72.2 (28.4)	63.2 (34.3)	71.9 (28.4)	60.9 (33.1)	54.9 (38.6)	75.0 (29.8)
% Of non-Hispanic other, mean (SD)	9.7 (17.0)	10.7 (20.3)	10.1 (16.5)	11.4 (19.2)	10.5 (22.9)	10.0 (19.8)
Clinical burden and complexity of a facility						
Mean Nosos score of patients, mean (SD)	1.6 (1.0)	1.5 (0.7)	1.6 (1.0)	1.6 (1.0)	1.7 (0.9)	1.6 (1.0)
VA facility complexity						
Most complex	7508 (60.2)	5548 (69.4)	7924 (60.3)	8049 (60.9)	4763 (64.2)	5922 (65.0)
Moderately complex	1969 (15.8)	1037 (13.0)	2136 (16.3)	2065 (15.6)	1121 (15.1)	1409 (15.5)
Least complex	1903 (15.3)	1125 (14.1)	2047 (15.6)	1993 (15.1)	1070 (14.4)	1128 (12.4)
Complexity score missing	1087 (8.7)	290 (3.6)	1029 (7.8)	1114 (8.4)	467 (6.3)	649 (7.1)
No. of patients per facility, mean (SD)	12 230.5 (18 946.7)	15 189.0 (21 299.3)	11 887.3 (18 747.9)	11 883.1 (18 657.1)	18 650.3 (22 583.6)	14 572.4 (20 798.8)
Type of VA facility						
VA hospital/inpatient facility	2256 (18.1)	1743 (21.8)	2301 (17.5)	2307 (17.5)	2251 (30.3)	1954 (21.5)
Community-based outpatient clinics	10 211 (81.9)	6257 (78.2)	10 835 (82.5)	10 914 (82.6)	5170 (69.7)	7154 (78.6)
Geographic characteristics of a facility						
Geographic region						
Northeast	2297 (18.4)	1233 (15.4)	2323 (17.7)	2394 (18.1)	1237 (16.7)	1404 (15.4)
Midwest	3116 (25.0)	1834 (22.9)	3249 (24.7)	3177 (24.0)	1528 (20.6)	2261 (24.8)
South	4421 (35.5)	3271 (40.9)	4623 (35.2)	4799 (36.3)	2824 (38.1)	3660 (40.2)
West	2633 (21.1)	1662 (20.8)	2941 (22.4)	2851 (21.6)	1832 (24.7)	1783 (19.6)
Urbanicity of a facility						
Urban	7292 (58.5)	5236 (65.5)	7533 (57.4)	7762 (58.7)	5313 (71.6)	5662 (62.2)
Rural	4069 (32.6)	2464 (30.8)	4524 (34.4)	4317 (32.7)	1633 (22.0)	2788 (30.6)
Missing	1106 (8.9)	300 (3.8)	1079 (8.2)	1142 (8.6)	475 (6.4)	658 (7.2)
Year of testing						
2006	673 (5.4)	213 (2.7)	766 (5.8)	747 (5.7)	414 (5.6)	411 (4.5)
2007	697 (5.6)	230 (2.9)	805 (6.1)	781 (5.9)	440 (5.9)	425 (4.7)
2008	757 (6.1)	263 (3.3)	841 (6.4)	813 (6.2)	488 (6.6)	473 (5.2)
2009	818 (6.6)	349 (4.4)	879 (6.7)	845 (6.4)	534 (7.2)	528 (5.8)
2010	858 (6.9)	438 (5.5)	928 (7.1)	893 (6.8)	574 (7.7)	560 (6.2)
2011	876 (7.0)	516 (6.5)	943 (7.2)	925 (7.0)	586 (7.9)	614 (6.7)
2012	900 (7.2)	581 (7.3)	945 (7.2)	956 (7.2)	593 (8.0)	684 (7.5)
2013	908 (7.3)	653 (8.2)	950 (7.2)	941 (7.1)	603 (8.1)	675 (7.4)
2014	933 (7.5)	706 (8.8)	954 (7.3)	978 (7.4)	609 (8.2)	735 (8.1)
2015	956 (7.7)	750 (9.4)	967 (7.4)	994 (7.5)	601 (8.1)	758 (8.3)
2016	964 (7.7)	778 (9.7)	979 (7.5)	1019 (7.7)	540 (7.3)	743 (8.2)
2017	1024 (8.2)	839 (10.5)	1061 (8.1)	1090 (8.2)	520 (7.0)	812 (8.9)
2018	1053 (8.5)	844 (10.6)	1058 (8.1)	1122 (8.5)	501 (6.8)	858 (9.4)
2019	1050 (8.4)	840 (10.5)	1060 (8.1)	1117 (8.5)	418 (5.6)	832 (9.1)

Abbreviatons: e-trigger, electronic trigger; FIT, fecal immunohistochemical test (FIT); FOBT, fecal occult blood test; IDA, iron-deficiency anemia; PACT, Patient-Aligned Care Team; Pi^2^, PACT Implementation Progress Index; VA, Veterans Affairs.

^a^ Mean value includes values from years 2011 to 2019 when PACT was implemented.

The study used 2 primary independent variables of interest. The first was year of testing, which was a 14-category variable with the first year, 2006, as the reference category. The variable indicated the year the tests with abnormal results were performed, and consequently, the year percentage of e-trigger positive flags and other facility variables were computed. The second variable, the Pi^2^ score, was a continuous variable that captured the extent of successful implementation of PACT by a facility in a year.^37,38^ This variable was computed centrally by the VA’s PACT Demonstration Laboratory Initiative group. The variable was estimated only for the years 2012 to 2017 and ranged from −8 to 8, with a higher value indicating a more successful PACT implementation. The composite score was determined using 8 core PCMH domains: (1) access, (2) continuity of care, (3) care coordination, (4) comprehensiveness, (5) self-management support, (6) patient-centered care and communication, (7) shared decision-making, and (8) delegation, staffing, and team functioning. Each facility was assigned an overall Pi^2^ score based on the number of domains in the top and bottom quartiles.^37^ The Pi^2^ was a time-varying variable, which captured the extent of PACT implementation for each facility across years. PACT implementation started during 2010 and there were no Pi^2^ scores before 2012 and after 2017, hence we used a value of −9 for years 2010 and before. Pi^2^ scores for 2012 were also used for 2011, and the scores for 2017 were also used for 2018 and 2019.

Other independent variables of interest included: (1) mean sociodemographic characteristics of patients in a facility, (2) clinical burden and complexity of a facility, and (3) geographic characteristics of a facility (Table 2). Mean sociodemographic characteristics of patients in a facility included: 1 continuous variable capturing the mean age of the patients; 2 continuous variables capturing the percentage of men and women; and 4 continuous variables capturing the percentage of patients who were Hispanic, non-Hispanic Black, non-Hispanic White, and non-Hispanic other.

Clinical burden and complexity of a facility included: 1 continuous variable capturing the mean Nosos score of the patients under study for each facility; 1 categorical variable capturing 4 VA facility complexity categories^39^ (most complex, moderately complex, least complex, missing); 1 continuous variable capturing the number of patients actively receiving any service at each facility during a year; and 1 categorical variable capturing 2 types of VA facilities (VA Hospital/Inpatient Facility and CBOCs). Geographic characteristics of a facility included: 1 categorical variable capturing the 4 US geographic regions (Northeast, Midwest, South, And West); 1 categorical variable capturing a facility’s urbanicity with 3 categories (urban, rural, and missing urbanicity).

### Statistical Analysis

We used retrospective observational cohort data with a panel data structure of more than 1000 facilities repeatedly observed over a 14-year period for each of the 6 diagnostic tests. Breusch and Pagan Lagrangian multiplier tests and Hausman tests were used to determine the appropriate panel data regression models.^40^ Based on these tests, the random-effects linear regression estimation was selected. The regressions were set up as a single-group time difference analysis, where the categorical year variable captured the before and after association between PACT implementation and percentage of e-trigger positive flags. A reduction in the percentage of e-trigger positive flags over time, especially during and after the years 2010 to 2012 when PACT was implemented, might indicate a potential improvement in the clinical processes due to fewer follow-up delays after PACT. All VA facilities were given access to similar resources, tools, and training to implement PACT at the same time (starting 2010).^25,41,42^ In addition to the year variable, the Pi^2^ score helped estimate the association between extent of PACT implementation^28,37,38^ and the percentage of e-trigger positive flags.

Stata statistical software versions 15.0 and 18.0 (StataCorp) were used for all the analyses from September 2021 and December 2023. *P* ≤ .05 was considered statistically significant a priori, and all statistical tests were 2-sided.

## Results

Over the 14-year study period, the study included the following tests with abnormal results in the form of 6 separate data sets: (1) 1 701 649 urinalysis tests performed on 808 148 patients; (2) 327 697 mammograms performed on 136 379 patients; (3) 1 378 765 FOBTs/FITs performed on 806 082 patients; (4) 2 067 274 laboratory test results suggestive of IDA performed on 390 911 patients; (5) 183 730 α-fetoprotein tests performed on 45 763 patients; and (6) 590 794 lung imaging tests performed on 391 098 patients. After aggregation of the diagnostic test–level and patient-level data, the urinalysis data had 1306 unique facilities contributing 12 467 facility-years of observations, the mammogram data had 1100 unique facilities contributing 8000 facility-years of observations, the FOBTs or FITs data had 1307 unique facilities contributing 13 136 facility-years of observations, the IDA test data had 1360 unique facilities contributing 13 221 facility-years of observations, the α-fetoprotein testing data had 1067 unique facilities contributing 7421 facility-years of observations, and the lung imaging data had 1141 unique facilities contributing 9108 facility-years of observations (Table 2).

Across the 14 years, the mean (SD) percentage of tests flagged as e-trigger positive were 55.5% (29.6%) for FOBT or FIT, 56.0% (28.7%) for IDA tests, and 45.4% (33.5%) for lung cancer imaging. The remaining tests had 30% or less e-trigger positive flags (Table 2). The mean Pi^2^ scores across facilities in all 6 diagnostic test data sets were less than 0, although the scores ranged from −8 to 8 in all data sets.

The mean patient age across facilities was predominantly above 60 years for most of the diagnostic testing data sets, with most patients tested being non-Hispanic White (Table 2). Most of the patients in the study data were men (≥77%), except in the mammogram data set, where 92% were women. Most of the VA facilities in all data sets were categorized as highly complex (>60%) and were a CBOC (70%-80%). Most facilities were located in the urban areas (approximately 60%-70%) and in the southern geographic region (approximately 35%-40%). The number of abnormal results increased over time in all 6 diagnostic test data sets.

Descriptive examination of the percentage of e-trigger positive flags over time (Figure) showed that the percentage of missed follow-ups for diagnostic tests with abnormal results decreased the most between 2010 and 2013 during the early years of PACT implementation, but increased again after 2013 for most tests except the α-fetoprotein tests. The adjusted regression results confirmed these findings (Table 3). For urinalysis, mammograms, FOBT or FIT, and IDA testing, the reduction in percentage of e-trigger positive flags was most pronounced around the time of PACT implementation (2010-2012) and was not sustained in the later years in the adjusted regressions. During the initial years of 2010 to 2013, percentage of potentially missed timely follow-ups decreased between 3 to 7 percentage points for urinalysis, 12 to 14 percentage points for mammograms, 19 to 22 percentage points for FOBT/FIT, and 6 to 13 percentage points for IDA tests. Percentage of e-trigger positive flags in α-fetoprotein tests showed no significant changes around the time of PACT implementation but decreased in later years. Lung cancer imaging tests were not found to have significant changes in percentage of e-trigger positive flags over the 14 years. Better Pi^2^ scores were statistically significantly associated with lower percentages of e-trigger positive flags for urinalysis (0.3–percentage point reduction [95% CI, −0.6 to −0.1] with 1-point increase in the score), and IDA tests (0.5–percentage point reduction [95% CI, −0.8 to −0.2] with 1-point increase in the score).

**Figure.**
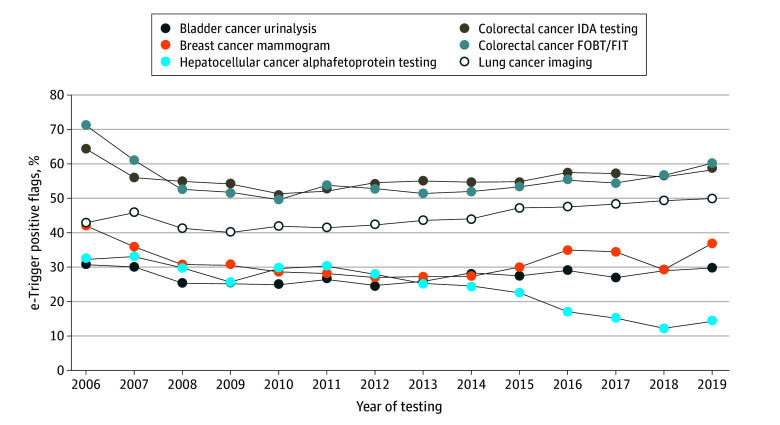
Change in Percentage of e-Trigger Positive Flags Over Time, 2006 to 2019 The figure depicts the trend over time of the e-trigger positive flags, with the percentage of e-trigger positive flags captured in the y-axis and the year of testing captured in the x-axis. FIT indicates fecal immunohistochemical test; FOBT, fecal occult blood test; IDA, iron deficiency anemia.

**Table 3.** Linear Regressions with Panel Data Random-Effect Adjustments Examining the Facility-Level Characteristics Associated with Percentage of e-Trigger Positive Flags

	Marginal effects, percentage points (95% CI)					
	Bladder cancer urinalysis (n = 12 467)	Breast cancer mammogram (n = 8000)	Colorectal cancer FOBT/FIT (n = 13 136)	Colorectal cancer IDA testing (n = 13 221)	Hepatocellular cancer α-fetoprotein Testing (n = 7421)	Lung cancer imaging (n = 9108)
**Level of PACT program adoption**						
Pi^2^ score	−0.3 (−0.6 to −0.1)	−0.2 (−0.6 to 0.1)	−0.002 (−0.27 to 0.26)	−0.5 (−0.8 to −0.2)	0.1 (−0.3 to 0.5)	0.1 (−0.2 to 0.5)
**Mean sociodemographic characteristics of patients in a facility**						
Mean age	0.11 (0.01 to 0.22)	0.3 (0.1 to 0.4)	0.8 (0.6 to 0.9)	0.9 (0.7 to 1.0)	−0.02 (−0.22 to 0.18)	−0.5 (−0.7 to −0.3)
% Of men	−0.04 (−0.08 to −0.01)	−0.04 (−0.08 to 0.01)	−0.03 (−0.09 to 0.03)	−0.12 (−0.15 to −0.08)	0.001 (−0.073 to 0.074)	−0.003 (−0.07 to 0.07)
% Of non-Hispanic White	−0.02 (−0.04 to 0.01)	−0.02 (−0.05 to 0.01)	0.03 (0.002 to 0.06)	−0.03 (−0.06 to −0.01)	−0.05 (−0.08 to −0.03)	0.03 (0.0003 to 0.07)
**Clinical burden and complexity of a facility**						
Mean Nosos score of patients	−2.9 (−3.7 to −2.1)	−1.5 (−3.4 to 0.4)	−3.9 (−5.1 to −2.8)	−3.2 (−4.3 to −2.2)	−2.9 (−4.2 to −1.6)	−2.6 (−3.9 to −1.3)
VA facility complexity						
Most complex	−3.5 (−6.0 to −0.9)	−7.9 (−11.2 to −4.5)	−6.2 (−9.1 to −3.3)	−3.1 (−5.5 to −0.7)	−0.5 (−4.0 to 3.0)	−0.4 (−3.9 to 3.1)
Moderately complex	−6.4 (−9.0 to −3.8)	−7.5 (−11.1 to −3.9)	−8.7 (−12.0 to −5.5)	−3.9 (−6.5 to −1.2)	−0.07 (−3.9 to 3.7)	−0.4 (−4.0 to 3.1)
Complexity score missing	−4.3 (−10.2 to 1.6)	0.7 (−8.9 to 10.2)	−4.7 (−12.3 to 2.9)	0.9 (−6.3 to 8.1)	5.1 (−6.0 to 16.2)	6.2 (−5.7 to 18.1)
No. of patients per facility	0.0001 (0.000 01 to 0.0002)	0.0001 (−0.000 005 to 0.0002)	−0.000 06 (−0.0001 to 0.000 03)	0.0001 (0.000 01 to 0.0002)	−0.000 009 (−0.0001 to −0.0001)	0.0001 (−0.000 02 to 0.000 17)
Type of VA facility						
Community-based outpatient clinics	9.3 (6.0 to 12.7)	−3.5 (−7.8 to 0.8)	3.8 (−0.5 to 8.1)	4.1 (0.8 to 7.5)	5.1 (1.2 to 8.9)	3.7 (−1.0 to 8.4)
**Geographic characteristics of a facility**						
Geographic region						
Midwest	−1.8 (−4.1 to 0.6)	−0.6 (−4.0 to 2.7)	4.2 (1.1 to 7.2)	−0.6 (−3.1 to 1.9)	−4.0 (−7.6 to −0.5)	−3.3 (−6.9 to 0.2)
South	3.1 (0.7 to 5.6)	2.8 (−0.6 to 6.1)	6.2 (3.1 to 9.2)	0.9 (−1.6 to 3.5)	1.0 (−2.4 to 4.4)	−2.7 (−6.2 to 0.7)
West	1.7 (−1.0 to 4.4)	2.3 (−1.3 to 6.0)	3.2 (−0.3 to 6.6)	−0.3 (−3.2 to 2.5)	1.7 (−2.0 to 5.4)	4.7 (0.6 to 8.9)
Urbanicity of a facility						
Rural	−2.4 (−4.3 to −0.5)	−1.5 (−3.9 to 0.8)	0.7 (−1.7 to 3.1)	−0.8 (−2.7 to 1.0)	−1.7 (−4.4 to 1.0)	2.2 (−0.3 to 4.8)
Missing	−7.0 (−13.2 to −0.8)	−4.6 (−14.2 to 5.0)	−5.8 (−13.2 to 1.6)	−12.6 (−20.0 to −5.3)	−14.2 (−24.2 to −4.1)	−12.6 (−24.1 to −1.1)
**Year of testing**						
2007	−1.8 (−4.4 to 0.9)	−7.5 (−13.6 to −1.4)	−10.2 (−12.3 to −8.1)	−7.6 (−10.2 to −5.0)	0.2 (−4.1 to 4.4)	1.9 (−2.3 to 6.0)
2008	−6.0 (−8.5 to −3.5)	−12.7 (−18.9 to −6.4)	−18.9 (−21.0 to −16.8)	−9.3 (−11.9 to −6.7)	−2.6 (−6.8 to 1.6)	−3.4 (−7.6 to 0.9)
2009	−6.6 (−9.2 to −4.0)	−12.2 (−18.2 to −6.2)	−20.2 (−22.4 to −17.9)	−10.3 (−13.0 to −7.6)	−7.5 (−11.4 to −3.5)	−4.9 (−9.0 to −0.7)
2010	−7.2 (−9.7 to −4.8)	−13.7 (−19.3 to −8.1)	−22.3 (−24.5 to −20.0)	−13.1 (−15.8 to −10.4)	−2.9 (−7.1 to 1.3)	−3.6 (−7.9 to 0.6)
2011	−3.1 (−6.4 to 0.2)	−12.1 (−18.2 to −5.9)	−18.8 (−22.0 to −15.5)	−7.2 (−10.6 to −3.7)	−3.4 (−8.8 to 2.0)	−4.8 (−10.1 to 0.4)
2012	−4.6 (−8.0 to −1.2)	−13.9 (−20.2 to −7.6)	−20.1 (−23.3 to −16.9)	−6.3 (−9.7 to −2.9)	−5.1 (−10.6 to 0.4)	−4.0 (−9.4 to 1.5)
2013	−3.6 (−7.0 to −0.2)	−14.1 (−20.3 to −7.9)	−21.6 (−25.0 to −18.2)	−5.5 (−9.1 to −1.9)	−7.6 (−13.1 to −2.1)	−2.4 (−8.0 to 3.3)
2014	−2.3 (−5.6 to 1.1)	−14.3 (−20.5 to −8.0)	−21.6 (−24.9 to −18.4)	−6.1 (−9.5 to −2.7)	−8.6 (−14.2 to −2.9)	−1.8 (−7.3 to 3.6)
2015	−3.2 (−6.5 to 0.2)	−11.5 (−17.8 to −5.1)	−21.6 (−24.9 to −18.2)	−7.5 (−10.9 to −4.2)	−11.2 (−16.7 to −5.6)	0.8 (−4.7 to 6.4)
2016	−1.8 (−5.1 to 1.5)	−6.7 (−13.0 to −0.4)	−19.0 (−22.3 to −15.7)	−4.9 (−8.3 to −1.6)	−16.3 (−21.9 to −10.6)	2.2 (−3.3 to 7.6)
2017	−3.7 (−7.1 to −0.3)	−7.8 (−14.0 to −1.6)	−20.9 (−24.1 to −17.7)	−5.8 (−9.2 to −2.5)	−18.7 (−24.3 to −13.0)	2.3 (−3.1 to 7.7)
2018	−0.7 (−4.1 to 2.7)	−12.3 (−18.4 to −6.1)	−17.9 (−21.2 to −14.6)	−5.4 (−8.8 to −2.0)	−19.9 (−25.7 to −14.0)	4.5 (−0.9 to 9.9)
2019	0.1 (−3.2 to 3.5)	−4.6 (−10.9 to 1.7)	−14.3 (−17.7 to −11.0)	−3.2 (−6.7 to 0.3)	−16.8 (−23.0 to −10.7)	5.7 (0.2 to 11.3)

Higher mean patient population age and type of VA facility being a CBOC were typically associated with higher percentages of e-trigger positive flags, whereas higher Nosos scores and facility complexity levels were associated with lower percentages of e-trigger positive flags (Table 3). Most other facility characteristics had no statistically significant association and/or directionally consistent significant association with e-trigger positive percentages across the 6 diagnostic test data sets.

## Discussion

This cohort study investigated whether PACT implementation was associated with an improvement in timely follow-up of abnormal test results in the VA health care system. Although PACT implementation was associated with a decrease in the percentage of potentially missed timely follow-ups for most tests with abnormal results, beneficial reductions were not sustained over time. In addition, better PACT implementation scores were associated with a decrease in potentially missed timely follow-up percentages only for certain tests.

Lack of timely follow-up of abnormal test results is often due to challenges associated with communication, care coordination, and effective teamwork. PCMHs aim to address these challenges by facilitating patient-centered, coordinated, comprehensive, accessible, high quality, and safe medical care.^43^ PACT emphasized strong and consistent communication between patients and clinicians, improved timely health care access for patients, and better coordinated team-based care to ensure that follow-ups and care transitions do not fall through the cracks. Most systematic reviews and large system-based studies have demonstrated positive improvement in quality of care, patient and caregiver experience, and reduced utilization due to PCMH implementation,^21,22,23,24,25,26,27^ however, none of these studies look at long-term outcomes, or focus on safety problems such as follow-up of diagnostic test results.

For instance, studies have found that the PACT implementation was associated with improvements in clinical processes, better quality of care, better patient satisfaction, lower staff burnout, better patient outcomes for chronic diseases, lower racial disparities, modest increase in primary care visits, and decrease in high-cost preventable inpatient stays and outpatient visits with mental health specialists.^37,41,42,44,45^ Past studies have also established that VA facilities that had higher extent of PACT components in place had greater improvements in several chronic disease quality measures as compared with VA facilities that had the lowest extent of PACT components in place. The improvements in chronic disease outcomes included better hemoglobin A_1C_, blood pressure, and blood cholesterol levels.^37,38,41^ To our knowledge, our study is the first to look at the association of PACT implementation and timely follow-up of diagnostic tests with abnormal results, which is particularly important in cancer where delayed diagnosis leads to considerable disease burden for patients and health systems. Our findings suggest that additional multicomponent and sustained interventions are needed to address problems related to missed follow-up of test results, which is an intractable problem to solve in most health systems and exists in the VA despite robust national guidelines for clinicians to follow-up on test results in a timely manner.^46^

### Limitations

Our study has certain limitations. First, the results may not be generalizable outside the VA; however, similarities between PACT and PCMH suggest broader applicability. Second, to better establish the association between PACT implementation and timeliness of abnormal diagnostic test follow-ups, it would have been ideal if at any given period the data contained VA facilities with and without PACT implementation, thereby facilitating a difference-in-difference comparison. This was not possible given the simultaneous PACT rollout across all VA facilities from 2010 to 2012. Third, the VA implemented the Veterans Access, Choice, and Accountability Act in 2014 and the Maintaining Internal Systems and Strengthening Integrated Outside Networks Act after 2014. These acts facilitated the use of services outside the VA. Consequently, the drop in the follow-up and slight increase in e-trigger positive flags after 2014 in some of the cancer diagnostic tests examined could be because veterans were accessing follow-up care outside the VA, which was not being captured by our data. Nevertheless, to our knowledge, this study is the longest assessment of the VA-based medical home model and timeliness of abnormal test follow-ups using 6 different types of cancer-related diagnostic tests. The long study period, the national-level data, and the extensively validated e-trigger algorithms by our team substantially improve both the internal validity and generalizability of this study.

## Conclusions

This cohort study found that PACT implementation was associated with an initial reduction in percentage of potentially missed timely follow-ups of abnormal test results for most cancer-related diagnostic tests. However, beneficial reductions were not sustained over time. Better PACT implementation was associated with a decrease in potentially missed timely follow-up percentage for some test results but not all. These findings suggest that PACT implementation had a short-term association with potentially improved quality of test result follow-up, but additional sustained interventions are required to prevent persistent care delays.

## Notes

Supplement.
Data Sharing Statement
