# Error in Table 2

**DOI:** 10.1001/jamanetworkopen.2024.9604

**Published:** 2024-04-08

In the Original Investigation titled “Medical Home Implementation and Follow-Up of Cancer-Related Abnormal Test Results in the Veterans Health Administration,”^1^ published March 14, 2024, there was an error in Table 2. A row label was incorrect and should have been labeled as “Mean sociodemographic characteristics of patients in a facility.” This article has been corrected.^1^
