# Supplementary material for: Medical Home Implementation and Follow-Up of Cancer-Related Abnormal Test Results in the Veterans Health Administration
Source: JAMA Netw Open. 2024 Mar 14;7(3):e240087. doi: 10.1001/jamanetworkopen.2024.0087 (PMC10940951; doi:10.1001/jamanetworkopen.2024.0087)
Supplement: Supplement. — Data Sharing Statement [file jamanetwopen-e240087-s001.pdf]

Data Sharing Statement

Rajan. Medical Home Implementation and Follow-Up of Cancer-Related Abnormal Test Results in the Veterans Health Administration. *JAMA Netw Open*. Published March 14, 2024. doi:10.1001/jamanetworkopen.2024.0087

Data
Data available: No

Additional Information
Explanation for why data not available: These data are from the Veterans' Affairs (VA) and the data sharing outside the VA health system is highly restricted.
